# Improved *piggyBac* Transformation with Capped Transposase mRNA in Pest Insects

**DOI:** 10.3390/ijms242015155

**Published:** 2023-10-13

**Authors:** Irina Häcker, Tanja Rehling, Henrik Schlosser, Daniela Mayorga-Ch, Mara Heilig, Ying Yan, Peter A. Armbruster, Marc F. Schetelig

**Affiliations:** 1 Department of Insect Biotechnology in Plant Protection, Justus Liebig University Giessen, Winchesterstr. 2, 35394 Giessen, Germanyh.schlosser@uq.edu.au (H.S.); ying.yan@agrar.uni-giessen.de (Y.Y.); marc.schetelig@agrar.uni-giessen.de (M.F.S.); 2 Liebig Centre for Agroecology & Climate Impact Research, 35394 Giessen, Germany; 3 Department of Biology, Georgetown University, 37th and O Streets NW, Washington, DC 20057-1229, USA; mch284@georgetown.edu (M.H.); paa9@georgetown.edu (P.A.A.)

**Keywords:** insect transgenesis, *Aedes*, *Drosophila suzukii*, tephritids, transformation efficiency, recombination efficiency, *piggyBac* transposase, helper plasmid, capped mRNA

## Abstract

Creating transgenic insects is a key technology in insect genetics and molecular biology. A widely used instrument in insect transgenesis is the *piggyBac* transposase, resulting in essentially random genomic integrations. In contrast, site-specific recombinases allow the targeted integration of the transgene construct into a specific genomic target site. Both strategies, however, often face limitations due to low transgenesis efficiencies. We aimed to enhance transgenesis efficiencies by utilizing capped mRNA as a source of transposase or recombinase instead of a helper plasmid. A systematic comparison of transgenesis efficiencies in *Aedes* mosquitoes, as models for hard-to-transform insects, showed that suppling *piggyBac* transposase as mRNA increased the average transformation efficiency in *Aedes aegypti* from less than 5% with the plasmid source to about 50% with mRNA. Similar high activity was observed in *Ae. albopictus* with *pBac* mRNA. No efficiency differences between plasmid and mRNA were observed in recombination experiments. Furthermore, a hyperactive version of *piggyBac* transposase delivered as a plasmid did not improve the transformation efficiency in *Ae. aegypti* or the agricultural pest *Drosophila suzukii*. We believe that the use of mRNA has strong potential for enhancing *piggyBac* transformation efficiencies in other mosquitoes and important agricultural pests, such as tephritids.

## 1. Introduction

Transgenic insect creation is an advancing technology crucial for molecular biology and insect genetics, employed for understanding gene functions and developing genetically modified strains for agricultural and vector control. The advent of CRISPR has accelerated its applications across multiple insect species, but the efficiency is often challenged by the need for a sequenced genome. Transposon-based transformation, like *piggyBac* (*pBac*) transposase, remains standard due to its independence from high-quality genome sequences [1,2,3,4,5,6]. It is applied universally across insects, including *Aedes* and *Anopheles* mosquitoes [7,8,9,10,11,12].

Transposon-based insect transgenesis involves a bipartite system of helper and donor plasmids injected into preblastodermal insect embryos, achieving germline transformation. The helper plasmid encodes the transposase gene, the donor plasmid the transgene construct flanked by inverted terminal repeat (ITR) sequences of the transposable element. Only the transgene construct plus the ITRs are inserted into the genome. Transposases’ short recognition sequences allow random integration of the transgene construct into the genome. While this is a versatile tool for gene function study [13,14], it can reduce the transformed lines’ fitness. Site-specific recombination (SSR) systems, like phiC31 integrase [15] and Cre recombinase [16], can address this, integrating the transgene construct into a previously introduced genomic “landing site” via recombinase-mediated cassette exchange (RMCE).

While species like *Drosophila melanogaster* have relatively high transposition and recombination efficiencies [17,18,19,20], others, notably mosquitoes, experience low efficiencies, often between 0% and less than 10% [2,7,8,9,21,22,23,24,25], making the creation of transgenic lines in non-model organisms challenging.

Higher transposition and recombination efficiencies in *Ae. aegypti* and other mosquito species would facilitate the application of transgenic work in this taxon of medically important insects. A widely used helper plasmid for *pBac*-mediated mosquito transformation drives transposase expression via the *D. melanogaster* heat shock promoter 70 (hsp70) [4]. We use the same promoter to drive recombinase expression in Cre and phiC31 helper plasmids [18]. One cause for the low efficiencies observed with these helper plasmids could be a low transcriptional activity of the exogenous *D. melanogaster* hsp70 promoter in *Aedes*, which could be solved by injecting capped mRNA instead of the helper plasmid. Additionally, a low activity of the enzyme itself could be responsible.

For *pBac* transposase, an alternative solution could be a hyperactive version of the *pBac* enzyme (hyPBase) selected in a *Saccharomyces cerevisiae* mutagenesis screen [26]. In the initial study, this hyperactive version did not improve the transposition efficiency in *Ae. aegypti* and *D. melanogaster* in genetic transformations [27]. However, a systematic comparison between the original *pBac* transposase and hyPBase, both under the control of the *D. melanogaster* hsp70 promoter, achieved up to 15-fold higher transposition efficiencies with hyPBase in *Tribolium castaneum*, *D. melanogaster*, and *Ceratitis capitata* [28].

Our study aimed to test this hsp70-driven hyperactive *pBac* version (*phsp-^i^hyPBase*) again in *Ae. aegypti* and additionally in the agricultural pest *D. suzukii* (Spotted Wing *Drosophila*, SWD) in comparison to the standard *phsp-pBac* helper plasmid. The second aim was to systematically compare the transformation efficiency of the hsp70 helper plasmids to that of in vitro transcribed, capped mRNA for *pBac* transposase, phiC31 integrase, and Cre recombinase in *Ae. aegypti*, and to test *pBac* and Cre mRNA efficiencies in the congeneric species *Ae. albopictus*. Our findings are compared and discussed in the context of potential variances in mRNA efficiency among different transposition and recombination mechanisms.

## 2. Results

### 2.1. ^i^hyPBase Helper Plasmid Does Not Increase the Transposition Efficiencies in Ae. aegypti and D. suzukii Embryos

To test if the original hyperactive version of the *pBac* transposase with the insect codon usage under the *D. melanogaster* hsp70 promoter (^i^hyPBase) [28] results in higher transposition efficiencies in the yellow fever mosquito, *Ae. aegypti*, embryos of the Higgs White Eye (HWE) strain were injected with varying concentrations of the *phsp-^i^hyPBase* helper plasmid in combination with two different donor plasmids, including a variation of helper–donor ratios. In parallel, the same injections were performed with the standard *phsp*–*pBac* plasmid [4]. Moreover, the results were compared to the transposition efficiencies obtained with the *phsp–pBac* plasmid in our laboratory over the years, which had also been performed at various helper concentrations and helper/donor ratios in the attempt to optimize *phsp–pBac*-mediated transposition efficiencies (Table 1 and Table S1). ^i^hyPBase helper plasmid concentrations were chosen lower than the *phsp–pBac* concentrations typically used in the expectance of higher transposition efficiencies as observed in *T. castaneum*, *D. melanogaster*, and *C. capitata* [28].

In the three injections with the ^i^hyPBase plasmid, the larval hatch rate of about 7–8% was markedly lower than typically observed in *phsp–pBac* injections (between 10 and 24%) (Table 1). However, this difference was statistically not significant (*p* = 0.081, single factor ANOVA). No noticeable difference could be observed for the adult emergence rate, and the fertility of the G_0_ families was comparable with both helper plasmids (Table 1 and Figure S1a–d; *p* = 0.175, and *p* = 0.647, single factor ANOVA). The average transposition efficiency in our laboratory with the *phsp–pBac* plasmid over the years was 1.46% (thirteen experiments in total, including seven experiments with no transgenic events). In this set of parallel injections with the ^i^hyPBase plasmid and *phsp–pBac*, no transgenic event was recovered (a total of more than 2200 injected embryos each, with 23,000 screened G_1_ for ^i^hyPBase and 34,000 screened G_1_ for *phsp–pBac*). 

The same ^i^hyPBase helper plasmid was tested for transposition efficiency in the agricultural pest *D. suzukii* and compared to previous injections with the standard *phsp–pBac* helper. In *D. suzukii*, the standard injection mix concentration for the *phsp–pBac* helper over the years was predominantly 200 ng/µL helper and 500 ng/µL donor plasmid. These concentrations were also used for ^i^hyPBase injections in two experiments. Moreover, both helper plasmids were mixed at equal concentrations in three independent injection experiments (Table 2). As in *Ae. aegypti*, the ^i^hyPBase did not improve the transformation efficiency compared to the standard *phsp–pBac* plasmid. We also did not observe differences in the development of injected embryos between *phsp–pBac* and *phsp-^i^hyPBase* injections (Table 2, Figure S1e–g). Notably, however, increasing the *phsp–pBac* helper and donor plasmid concentrations to 300 ng/µL and 700 ng/µL, respectively, improved the transformation efficiency for two of three donor constructs (compare injections Ds15/Ds18, Ds16/Ds19, Ds17/Ds20).

### 2.2. pBac mRNA Boosts the Transposition Efficiency in Ae. aegypti

To assess the efficiency of in vitro transcribed, capped mRNA as a *pBac* helper source, *pBac* mRNA injections were performed with six different donor plasmids (V19, V96, V97, V368, V369, and V370) ranging from 7 to almost 12 kb in plasmid size and 3.6 to 6.5 kb in insert size. The minimal transposition efficiencies of these injections were compared to the *phsp–pBac* helper plasmid injections performed over the years in our laboratory with various donor plasmids (Table 3 and Table S2). Of the six donor plasmids used in *pBac* mRNA injections, V19 had previously been injected with the *pBac* helper plasmid. Moreover, the V370 donor plasmid used in mRNA injections is identical to the V258 donor injected with *pBac* helper plasmid except for an additional *attB* recombination site in V370. As all the plasmid helper and mRNA helper experiments were conducted at different time points, with different HWE female cohorts, by different experimenters, and some in a different laboratory (V369, V370), we also performed side-by-side injections with the *phsp–pBac* plasmid or *pBac* mRNA together with the V368 donor plasmid into embryos collected from the same batch of HWE females.

Injection numbers across all experiments ranged from 150 to more than 1000 injected embryos per experiment for the helper plasmid and from 110 to less than 600 embryos per experiment for the helper mRNA (Table 3 and Table S2). In helper plasmid injections, *pBac* and donor concentrations varied between 160 and 400 ng/µL and 150 and 600 ng/µL, respectively. In mRNA injections, the donor concentration was kept constant at 300 ng/µL, and the mRNA concentration varied between 100 and 300 ng/µL. G_0_ injection survivors were backcrossed individually or in groups of up to 15 individuals, depending on the number of G_0_ survivors and the current insectary capacity, and the offspring (G_1_) screened for fluorescent marker expression. For all mRNA injections and three of the *pBac* plasmid injections (exp. 2, 3, 9), a subset of positive G_1_ of each G_0_ family was analyzed for the transgene copy number in the genome via droplet digital PCR (ddPCR). In the case of single integration events identified by ddPCR, inverse PCR was performed to determine and distinguish between genomic integration sites. Some individuals were sacrificed for the transformation event analysis only after individual backcrossing and successful mating. The minimum number of independent genomic integration events per G_0_ family was determined by summing up the number of G_1_ with a different copy number or integration sites. A copy number greater than one was counted as one independent event in this analysis. The complete set of data for the integration event analysis is summarized in Table S3.

**Figure 1:**
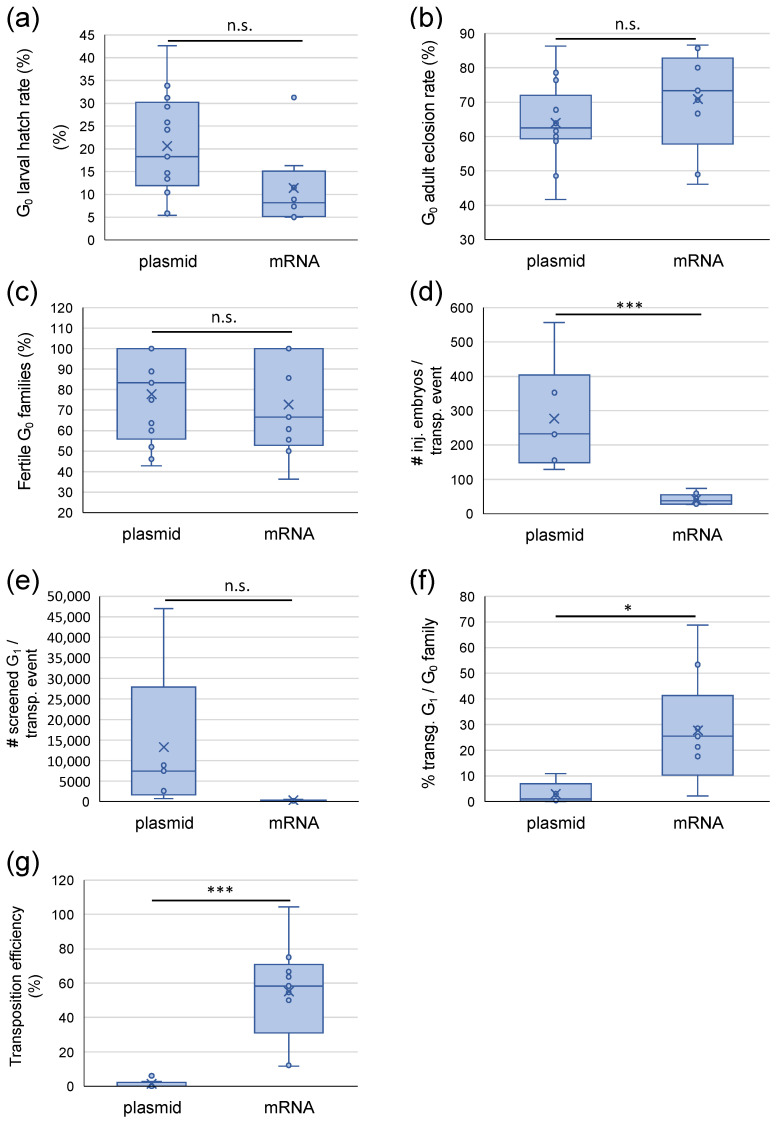
Effect of *pBac* helper plasmid and *pBac* mRNA injections in *Ae. aegypti* on larval hatch rate (**a**), G_0_ adult eclosion rate (**b**), percentage of fertile G_0_ families (**c**), the number of injected embryos per transposition event (**d**), the number of screened G_1_ per transposition event (**e**), the percentage of transgenic G_1_ per positive G_0_ family (**f**), and the minimal transposition efficiency (%) (**g**). Box and whisker plots (exclusive median) are displayed based on the data presented in Table 3 and Table S2. The horizontal line represents the median; the cross (x) represents the mean; n.s. = no significant difference, *p* > 0.05; * = significant difference, *p* ≤ 0.05; *** = significant difference, *p* ≤ 0.001.

Across all injections, we observed a lower but not significantly different larval hatch rate in the mRNA helper injections (Figure 1a; *p* = 0.06619, one-factor ANOVA), which seemed independent of the size of the injected donor construct. There was no difference in the G_0_ adult eclosion rate and in the fertility of the G_0_ families between plasmid and mRNA helper (Figure 1b,c; *p* = 0.2340 and *p* = 0.6204, respectively, one-factor ANOVA). However, the transposition efficiency increased on average from 1.49% obtained with the helper plasmid to more than 50% with the mRNA helper (Figure 1g, *p* = 1.646 × 10^−6^). These numbers refer to the calculated minimal transposition efficiency, i.e., the number of obtained independent transgenic events divided by the total number of adult G_0_ survivors of each injection. Different independent events within one G_0_ family, as determined by digital PCR and inverse PCR, were counted separately. The actual transposition efficiency, however, in several cases, might have been even higher than the numbers reported here for the following reasons: (i) G_0_ injection survivors were not always backcrossed individually (Table S2). Thus, in some cases, more than one positive G_0_ could have been in a positive pool, resulting in more than one independent transgenic event, which ddPCR and inverse PCR would not have necessarily identified, as only small numbers of G_1_ individuals per family were assessed. This especially applies to the V369 and V370 injections (exp. 16, 17), the only mRNA injection experiments where the G_0_ were exclusively backcrossed in large groups (12 individuals per cage, see Table S3). Moreover, of several G_0_ families, the offspring was not molecularly characterized by copy number and integration sites. (ii) All emerged G_0_ adults were counted for the calculation, including sterile individuals, which, on average, were about 40% (Table S4). This G_0_ sterility rate is comparable to published numbers for *Ae. aegypti* [24,25].

A frequent observation with *pBac* mRNA injections was multiple transgene construct integrations in one individual, ranging from two to eighteen integrations at the higher mRNA concentration and two to fourteen integrations at the lower concentration in the G_1_ animals analyzed by ddPCR. Different integration frequencies were observed between the offspring of different G_0_ founders and within the offspring of single-founder G_0_ families, including individuals with single integrations at different genomic locations as determined by inverse PCR. On the contrary, from all *phsp–pBac* plasmid injections, only one individual with two integrations of a transgene construct was obtained. For the detailed results, see Tables S2 and S3.

### 2.3. Preliminary Data Indicate High Transposition Efficiencies with pBac mRNA in Ae. albopictus

The efficiency of capped *pBac* mRNA was also assessed for *Ae. albopictus*. In this experiment, no control injections with *pBac* helper plasmid were performed, as the primary purpose was to create landing site lines for RMCE. G_0_ survivors were exclusively backcrossed in groups of 10–15 individuals, and the positive G_1_ offspring was not further analyzed molecularly to identify different transposition events within a G_0_ family. Nevertheless, this experiment indicated a similarly high transposition rate in *Ae. albopictus* as in *Ae. aegypti*, as 15 of the 19 G_0_ families produced positive G_1_ (Table 4). This percentage of positive families is comparable to the *Ae. aegypti* mRNA injection experiments 16 and 17, where G_0_ were backcrossed in a similar scheme (mostly groups of 12). These group backcrosses produced a similar rate of positive families, i.e., eleven of twelve (exp. 16) and five of six families (exp. 17) (Table 3 and Table S2). If only the number of positive families is counted as independent transposition events in these experiments, this results in a transposition efficiency of 7%, i.e., in the same range as observed for the *Ae. albopictus* mRNA injection (6.3%). We, therefore, assume that the actual transposition efficiency in *Ae. albopictus* was much higher.

### 2.4. Capped mRNA Does Not Improve Cre- or phiC31-RMCE Efficiencies in Aedes

Based on the strong positive effect of capped mRNA on *pBac* transposition efficiencies, we subsequently tested the efficiency of capped mRNA in phiC31- and Cre-RMCE experiments in direct comparison to the respective helper plasmid.

For phiC31-RMCE, comparative injections were performed at two different helper/donor concentrations, 150/300 ng/µL and 300/500 ng/µL, in *Ae. aegypti*. Concentrations were chosen to be in the same range as concentrations used in phiC31-RMCE experiments in two different landing site lines previously performed in our laboratory (Table S6). They were also comparable to the *pBac* mRNA concentrations. Five independent injections were performed for the lower concentration, two with the phiC31 helper plasmid and three with the phiC31 mRNA. For the higher concentration, two replicates each were performed (Table 5). No significant differences in the larval hatch rate, adult eclosion rate, or G_0_ fertility were observed between helper plasmid and mRNA injections (*p* = 0.109, *p* = 0.181, *p* = 0.456, respectively; statistics conducted across all concentrations; see Figure S2). Based on the numbers obtained with *pBac* mRNA, one transgenic event would have been expected for about every 50th injected embryo, assuming a similar efficiency. However, the overall recombination efficiency was very low. Only in one of the three replicates with mRNA at the lower helper/donor concentration we observed one RMCE event. In comparison, we identified one donor plasmid integration event in one of the helper plasmid injections at the higher helper/donor concentration (Table 5). The corresponding recombination efficiencies within the replicates were 10 and 6.45%, respectively, and 3.34 and 3.23% across the replicates.

The efficiencies obtained with the helper plasmid were in the same range as those observed in the previous experiments, performed with the same or different donor constructs into different landing site lines (Table S6, exp. I–V).

In phiC31-RMCE injections, the positive effect of the mRNA could not be observed when mRNA concentrations similar to the *pBac* transformations were used. Thus, concentrations were increased for Cre-RMCE injections to 450 ng/µL for the helper (plasmid or mRNA) and 350 ng/µL for the donor plasmid. Helper plasmid and mRNA injections were performed in three independent replicates each. Similar to the phiC31 experiments, only one plasmid and mRNA replicate produced recombination events. In the positive plasmid injection replicate, one family produced an RMCE phenotype, and another one offspring with a donor plasmid integration phenotype, corresponding to an overall minimum recombination efficiency of 2.44% (0.8% across all replicates, Table 6). In the mRNA injections, a single family produced offspring with an integration phenotype, corresponding to a minimum recombination efficiency of 3% (1% across all replicates). For complete injection, crossing, and screening data, see Table S7.

We also performed Cre-RMCE injections in *Ae. albopictus* into two different landing site lines. In one line (17A1), we tested injecting with either 150 ng/µL *phsp-CRE* or 190 ng/µL helper mRNA. From 34 fertile families of G_0_ founders injected with helper plasmid, one showed expression of the donor transgene in addition to the original landing site marker, indicating an integration event. This translates into a recombination efficiency with a Cre helper plasmid of 2.27% (Table S8). None of the 46 fertile families made of G_0_ founders injected with helper mRNA showed expression of the transgene.

In a second line (1A3), the landing site integrated 1.3 Mb upstream of the Nix locus as determined via inverse PCR, and marker fluorescence was only observed in males. Thus, all injected embryos were heterozygous for the landing site. Notably, in this line, the *Ae. aegypti* PUbeCFP expression was not visible, but PCR could confirm the presence of the sequence. This line was only injected with 150 ng/µL *phsp–CRE*, and one showed expression of the donor plasmid fluorescence marker from a total of six fertile families. PCR of the animals that showed transgene expression suggested both excision of the original PUbeCFP sequence and multiple, potentially tandem, integrations of the plasmid, i.e., non-canonical recombination events. 

## 3. Discussion

Producing transgenic insects can be time-consuming because transgenesis efficiency can be very low in many insect orders and species. This is particularly true for *piggyBac* transformation and RMCE experiments in *Aedes* mosquitoes. In many experiments published to date, the *phsp–pBac* helper plasmid [4] was used as a source of *pBac* transposase with the *D. melanogaster hsp70* promoter to drive transposase expression. Minimum transposition efficiencies in these publications are reported to be between 0 and 4% [7,24,25,29]. This transposition rate does not improve when the embryos are heat-shocked shortly after injection [24]. A large meta-analysis of *pBac* transposition efficiencies reports an average of 7% for *Ae. aegypti* (46 experiments, 3314 total G_0_ adults, 239 independent transgenic lines) and 1.6% for *Ae. albopictus* (10 experiments, 5339 G_0_ adults, 89 independent lines). Ways to improve the efficiencies would be desirable to reduce this bottleneck in transgenesis projects.

In attempts to improve transformation efficiencies in *Ae. aegypti* and *D. suzukii*, a hyperactive version (^i^hyPBase) of *pBac* transposase helper plasmid was tested. However, the results showed no improvement in either species. In *Ae. aegypti*, the lack of success could be due to lower donor and helper plasmid concentrations compared to a successful study by Eckermann et al. [28]. Additionally, the backcrossing of G_0_ adults may have masked the presence of transgenic G_0_. On the other hand, these factors did not apply to the *D. suzukii* injections. Our results in *Ae. aegypti* were consistent with those of Wright et al., who also did not observe transposition events with ^i^hyPBase in *Ae. aegypti*. However, we did not observe the G_0_ sterility reported by Wright et al. for both *Ae. aegypti* and *D. melanogaster* [27], and also no significant influence on other life parameters

We hypothesized that the exogenous *D. melanogaster hsp70* promoter does not function well in *Aedes* mosquitoes, causing low transposase levels. Therefore, we performed *pBac*-mediated transformations using in vitro transcribed, capped mRNA, resulting in a more than 30-fold increase in efficiency on average. Thus, while hundreds of embryos had to be injected with the *pBac* helper plasmid to obtain one transgenic line, with the mRNA a few dozen embryo injections were sufficient. These data are based on the results obtained with two different *Ae. aegypti* strains, HWE, and Orlando, six different donor plasmids, three different mRNA concentrations, and different injection personnel. It is important to note that the efficiencies reported in this work are minimal, as the original purpose of the experiments was not to determine transposition efficiencies. Therefore, G_0_ adults were backcrossed in groups to a certain extent in each injection. The actual efficiencies are probably higher, especially in mRNA injections 16 and 17, where all G_0_ individuals were backcrossed in large groups, and only a fraction of the positive offspring was analyzed molecularly. Moreover, for all efficiency calculations, the total number of G_0_ was considered, not the number of fertile G_0_.

The results of the capped *pBac* mRNA injections in *Ae. albopictus* strongly suggest a similarly high activity of capped mRNA in *Ae. albopictus* as in *Ae. aegypti*. Even though the experiment did not include individual G_0_ backcrosses and the positive G_1_ were not further assessed for integration copy numbers and integration sites, the high percentage of positive families is similar to that obtained in the *Ae. aegypti* injections 16 and 17, where we used a very similar G_0_ group backcrossing scheme (mostly 12 G_0_ per cage). Such a high percentage of positive families was not observed in any *pBac* helper plasmid injection in *Ae. aegypti*. If only the number of positive families is considered as the number of independent events for the transposition efficiency calculation of the *Ae. aegypti* injections 16 and 17, the transposition efficiency would be between 6 and 7%, i.e., identical to the one obtained for *Ae. albopictus* using this calculation.

One effect of mRNA-mediated *pBac* transformation in *Ae. aegypti* was the occurrence of multiple genomic copies of the transgene construct in many G_1_ individuals, as determined by ddPCR. Therefore, with *pBac* mRNA injections, it is recommended to conduct single backcrosses of at least some of the positive G_1_. Analyzing the copy number after line establishment is necessary to identify lines with a single integration event. One approach to minimize the frequency of multiple genomic insertions would be to titrate the mRNA concentration to optimize the fraction of G_1_ carrying only one genomic integration. We did not observe an apparent correlation between the mRNA concentration and the max. number of integrations per individual or the relative frequency of G_1_ with more than one integration. However, a precise analysis of a possible correlation was not possible, as in mRNA injections 16 and 17, the positive individuals were group backcrossed until G_2_. Molecular analysis of integration events was only performed with generation G_3_. During the two generations of backcrossing, integrations might have segregated, consistent with the overall low number of integration events per individual observed in these two injections compared to the other experiments.

Transposition efficiencies with *pBac* mRNA observed in our hands were substantially higher than the numbers reported for *Aedes* mosquitoes in the literature, ranging between 1 and 3.6% [2,9,21]. We can only speculate about the reason for the differences in efficiencies between published work and our study. Potentially, at very high mRNA concentrations (700 ng/µL [21]), too many genomic integrations occurred to result in a sufficient number of viable germ cells. Additionally, the nature of the donor construct, the strain’s genetic background, and the number of generations the strain had been cultivated in the lab could influence the transposition efficiency. While we injected into HWE and Orlando laboratory strains, the experiments by Labbe et al. and Haghighat-Khah et al. were performed in strains of Malaysian origin [9,21].

Injections using phiC31 capped mRNA for RMCE in *Ae. aegypti* have been published before, resulting in RMCE efficiencies between 0 and 5% in three different landing site line injections [21]. Our study included parallel helper plasmid injections into embryos from the same female cohorts to directly compare the recombination efficiencies with plasmid helper and mRNA helper. Based on the high transposition efficiencies observed with *pBac* mRNA, it was unexpected that efficiencies for RMCE experiments were not increased using mRNA as phiC31 or Cre recombinase source. The phiC31-RMCE efficiency of 10% achieved in one of the injections is in the same range as the published efficiencies for *Ae. aegypti* [21], although at about 5-fold lower mRNA concentration. 

The reasons for the lack of an mRNA effect in RMCE experiments in *Ae. albopictus* and *Ae. aegypti* are currently not known. We can exclude the inactivity of the injected mRNA due to degradation because leftover injection mixes from the injection needles were recovered and run on a gel to confirm mRNA integrity. One big difference between *pBac*-mediated transposition and Cre- or phiC31-mediated recombination is the lower frequency of sites in the genome at which insertions can occur. *pBac* transposase uses TTAA sequences in the genome for insertion, which are predicted to occur every 256 base pairs. In contrast, the recombination sites for Cre and phiC31 do not occur naturally in *Aedes* genomes. Thus, the genomes of our landing site lines contain exactly one position where recombination can take place, and the rate-limiting step might be the likelihood of the enzyme and donor plasmid being present at this position simultaneously. This likelihood can be increased by increasing the amount of injected mRNA and donor plasmid. However, this would be in the range of a maximum two- to three-fold increase, as the viscosity of the nucleic acid solution limits the injectability. Moreover, genomic excision experiments published previously by us and others show that in the case of Cre recombinase, the amount of Cre enzyme in the commonly used injection concentrations is not limiting, as excision efficiencies between 25% and 100% were obtained when both recombination sites were in proximity [21,29,34]. 

Another possibility would be the silencing of the landing site construct by heterochromatic factors. A recent study in *D. melanogaster* suggests, for example, that endogenous genes and exogenous reporter genes are differently regulated in areas of transcriptionally active constitutive heterochromatin, resulting in the silencing of P-element reporter, despite the close proximately to actively transcribed genes [35]. Interestingly, the same group also found that P-elements could be remobilized despite the reporter gene being silenced at the heterochromatic insertion site [35,36]. In our case, the high efficiency of the excisions, as well as the strong expression of fluorescent landing site reporters, argues against the possibility of heterochromatic factors silencing the landing site constructs. However, the apparent high complexity of heterochromatic organization and regulation makes it difficult to predict how transgene inserts will behave.

We speculate that the reason for the missing effect of the mRNA in RMCE injections might be the limited likelihood of co-localizing all three components for RMCE (landing site, enzyme, and donor plasmid) at the same time at a single site in the genome or kinetic and thermodynamic aspects of the RMCE reaction as discussed in [29]. On the other hand, the same situation applies to other insect species, such as *D. melanogaster*, *D. suzukii*, and *Anastrepha suspensa*, in which RMCE efficiencies between 10 and 20% were achieved (single experiments, [18,20,37,38]).

Nevertheless, for Cre-RMCE, we obtained the first one-step RMCE published so far in *Ae. aegypti*. In previous experiments, we only achieved two-step Cre-RMCE, obtaining integration lines first, from which, by injection of only Cre helper plasmid, the complete RMCE event was obtained [29].

## 4. Materials and Methods

### 4.1. Insect Rearing

*Ae. aegypti* wild-type strains and transgenic lines were reared in an insectary at constant conditions of 27 °C, 70% RH, and a 12:12 h light:dark cycle. Larvae were fed on Tetra TabiMin fish food pellets (Tetra GmbH, Melle, Germany). The adult mosquito diet was sterile-filtered 10% (*w*/*v*) sucrose solution. Moreover, adult females were fed once per week with pig blood purchased from a butcher shop. *Ae. aegypti* laboratory strains used in the experiments were the Orlando wild-type strain and the Higgs White Eye (HWE) strain (a spontaneous white eye mutant strain of the Rexville D strain from Puerto Rico [39]).

A lab colony of *Ae. albopictus* was established with pupae and larvae collected from an auto-salvage yard in Manassas, Virginia, in 2018. Animals were reared under standard laboratory conditions at 21–26 °C, 80% RH, 16 h light:8 h dark for three generations prior to injections for *pBac*-mediated transformation [40]. Larvae were fed on a Monday-Wednesday-Friday schedule with 1 mL of a larval food slurry consisting of 1 L DI water, 120 g dog food (Nutro Ultra Small Breed Puppy, Nutro Products Inc., Franklin, TN, USA), and 40 g frozen brine shrimp (Sally’s Frozen Brine Shrimp, San Francisco Bay Brand, Newark, CA, USA) [41]. Adult females were provisioned with organic raisins (Newman’s Own, Westport, CT, USA) to allow ad libitum sugar feeding. They were allowed to blood feed on a human host for egg production. The Georgetown University Institutional Review Board (IRB) has determined that mosquito blood feeding is not human research and does not require IRB approval; however, the blood feeding protocol has been approved by the Georgetown University Office of Health and Safety.

The wild-type *D. suzukii* USA strain and transgenic lines were maintained at 25 °C and 55–60% humidity with a 12 h photoperiod. Flies were briefly anesthetized with CO_2_ for screening and to set up crosses.

### 4.2. In Vitro Transcription (IVT) of pBac, phiC31, and Cre mRNA for Injections

#### 4.2.1. Production of the IVT Templates

*pBac* IVT template was obtained by PCR on the *phsp–pBac* plasmid [4], using the forward primer P1269 (5′GAAACTAATACGACTCACTATAGGGAGAGCCGCCACatgggtagttctttagacgatg; upper case letters represent T7 initiation sequence and linker) and the reverse primer P1270 (5′cttattagtcagtcagaaacaac). The PCR reaction contained 2 ng plasmid DNA, 500 nM of each primer, 200 µM of each dNTP, 1x Q5 reaction buffer, and 1 µL Q5 Polymerase (New England Biolabs, Ipswich, MA, USA ) in a final volume of 100 µL. The reaction was run in a BIO-RAD C1000 Touch Thermal Cycler (BIO-RAD, Hercules, CA, USA) (initial denaturation at 98 °C for 30 s, 30x [98 °C for 10 s; 51 °C for 20 s; 72 °C for 1 min] followed by the final elongation at 72 °C for 2 min).

Cre and phiC31 IVT templates were obtained by PCR on plasmids AH445 (*phsp–Cre*) and AH444 (*phsp–phiC31*), respectively. Primers were P2203 (5′GAAACTAATACGACTCACTATAGGGAGAGCCGCCACatgtccaatttactgaccgtacacc) and P2204 (5′gctaatcgccatcttccagcag) for Cre, and P1630 (5′GAAACTAATACGACTCACTATAGGGAGAGCCGCCACatggacacgtatgccggtgcttac) and P1631 (5′ctaggccgctacgtcttcggtgc) for phiC31. The PCR reaction contained 10 ng plasmid DNA, 500 nM of each primer, 100 µM of each dNTP, 1x Platinum Taq reaction buffer, 1.25 mM MgCl2, and 1 µL Platinum Taq DNA Polymerase (Life Technologies, Carlsbad, CA, USA) in a final volume of 50 µL. The reaction was run in a BIO-RAD C1000 Touch Thermal Cycler (initial denaturation at 95 °C for 2 min, 35x [94 °C for 30 s; 59 °C for 30 s; 72 °C for 2 min] followed by the final elongation at 72 °C for 10 min).

The PCR products were analyzed and purified by 1% agarose gel electrophoresis and gel extracted with the ZymoClean Gel DNA Recovery kit (Zymo Research Europe GmbH, Freiburg, Germany) according to the manufacturer’s instructions. 

#### 4.2.2. In Vitro Transcription Reaction and mRNA Purification

IVT was performed using the HiScribe T7 Arca mRNA kit (#2060S, NEB, Ipswich, MA, USA) according to the manufacturer’s instructions, using 800–1000 ng IVT template. mRNA was purified using the MegaClear Transcription clean-up kit (AM1908, Thermo Fisher Scientific, Waltham, MA, USA) according to the manufacturer’s instructions, choosing elution option 1 (50 µL elution solution on the column, incubate at 65 °C for 5 min) performed twice, and including the optional Ammonium Acetate precipitation. mRNA quality was analyzed by agarose gel electrophoresis, and mRNA was stored in 5 µL aliquots at −80 °C until use.

### 4.3. Preparation of Injection Mixes

Transposase/recombinase-encoding helper plasmids, or in vitro transcribed, capped mRNA were mixed with the corresponding donor plasmids at the final concentrations specified in Table 1, Table 2, Table 3, Table 4, Table 5 and Table 6 in RNAse-free 1x embryonic injection buffer (5 mM KCl, 0.1 mM NaPO_4_, pH 6.8). To remove particles and dust that could clog the injection needles, the injection mixes were centrifuged at 13,000 rpm for 30 min at 4 °C. The supernatant was taken carefully without disturbing a possible pellet and stored in 5 µL aliquots at −80 °C until further use.

### 4.4. Embryonic Microinjections of Ae. aegypti 

Injections for *pBac*-mediated transformation: *Ae. aegypti* transgenic lines were created by injecting preblastodermal embryos of the HWE strain with the *phsp*-*pBac* helper plasmid [4] or in vitro transcribed, capped *pBac* mRNA, and a donor plasmid at varying concentrations (see Table 1 and Table 3), in 1x embryonic injection buffer (EIB; 5 mM KCl, 0.1 mM NaPO4, pH 6.8). Injections were performed with a MN-151 micromanipulator (Narishige, Tokyo, Japan), a SZX16 stereo microscope (Olympus Life Science/Evident Europe GmbH, Hamburg, Germany), and pre-siliconized quartz injection needles prepared with a P-2000 laser puller (Sutter Instruments, Novato, CA, USA) from glass capillaries (Q100-70-7.5, Science Products GmbH, Hofheim am Taunus, Germany). Injected embryos were kept moist for two days to allow completion of embryonic development before being transferred to water with some TabiMin fish food for hatching. Survivors were sexed in the pupal stage and backcrossed to HWE in small groups or individually. G_1_ offspring were collected for 3–6 gonotrophic cycles and screened for the presence of the transgenic marker (DsRed or eGFP fluorescent protein) at the larval or pupal stage. Positive G_1_ were again backcrossed individually or in groups to establish transgenic lines.

Injections for recombinase-mediated cassette exchange: preblastoderm embryos of the *Ae. aegypti* landing site lines for phiC31-RMCE (lines V19-M2M1, V19-M26M3m2; V19 landing site construct = attP_3xP3-eGFP_attPrev) or Cre-RMCE (line V3-M30M1; V3 landing site construct = FRT_3xP3-DsRed_FRT3_loxN_3xP3-FRT5_AmCyan-lox2272-loxP_attP220rev) were injected with the recombinase helper plasmid or in vitro transcribed, capped recombinase mRNA and a donor plasmid at varying concentrations (see Table 5 and Table 6), in 1x EIB. Further rearing and crossing were identical to the *pBac* injections described above.

### 4.5. Embryonic Microinjections of Ae. albopictus

Injections for *pBac*-mediated transformation: *Ae. albopictus* transgenic lines were created by injecting preblastodermal embryos with in vitro transcribed, capped *pBac* mRNA (300 ng/µL) and the donor plasmid *pBXLII_FRT_3xP3DsRed_FRT3_loxN-PUbeCFP-lox2272* (150 ng/µL) in 1x EIB. Injections were performed with an MP-845 micromanipulator controlled by the Trio MPC-100 (Sutter Instruments, Novato, CA, USA) on an SZX12 stereo microscope (Olympus Life Science/Evident Europe GmbH, Hamburg, Germany) and pre-siliconized borosilicate injection needles were pulled with a P-2000 laser puller (Sutter Instruments, USA) from glass capillaries (cat #18100-3, World Precision Instruments, Sarasota, FL, USA). The microneedles were further sharpened by beveling with a BV-10 beveler (Sutter Instruments, USA).

Injected embryos were kept moist for two days to allow completion of embryonic development before transferring to water with three droplets of larval food slurry (described in *Ae. albopictus* insect rearing above). Survivors were sexed at the pupal stage and backcrossed to WT individuals in small groups of 10–15. G_1_ offspring were collected for 2–3 gonotrophic cycles and screened for the presence of the transgenic marker (DsRed or eCFP fluorescent protein) at the larval or pupal stage. Positive G_1_ were again backcrossed individually to establish transgenic lines.

Injections for recombinase-mediated cassette exchange: pre-blastoderm embryos of an *Ae. albopictus* landing site line for Cre-RMCE (line 17A1; landing site construct = *pBXLII_FRT_3xP3DsRed_FRT3_loxN-PUbeCFP-lox2272*) were injected with either the recombinase helper plasmid (150 ng/µL) or with in vitro transcribed, capped recombinase mRNA (190 ng/µL) and a donor plasmid (250 ng/µL) in 1x EIB. Survivors were sexed at the pupal stage and backcrossed to WT individuals (see the Manassas, VA collected population described above) individually or in small groups of 1–3. G_1_ offspring were collected for 2–3 gonotrophic cycles and screened for the presence of the transgenic marker (3xP3-AmCyan) at the larval or pupal stage. Positive G_1_ individuals were backcrossed individually.

An additional *Ae. albopictus* transgenic line (1A3) with a sex-linked landing site (the landing site integrated 1.3 Mb upstream of the *Nix* locus as determined via inverse PCR and was only observed in males) was injected with recombinase helper plasmid (150 ng/µL) and a donor plasmid (250 ng/µL) in 1x EIB. Survivors were sexed at the pupal stage and backcrossed to WT individuals individually or in small groups of 4–12. G_1_ offspring were collected for two gonotrophic cycles and screened for the transgenic marker (3xP3AmCyan) at the larval or pupal stage. Positive G_1_ were again backcrossed individually.

All *Ae. albopictus* injections were performed by the staff of the University of Maryland Institute for Bioscience & Biotechnology Research Insect Transformation Facility (Rockville, MD, USA)

### 4.6. Embryonic Microinjections of D. suzukii

Germline transformation with *piggyBac* constructs was carried out as previously described [6]. A mixture of the *piggyBac* donor construct (500 or 700 ng/µL) and the *phsp*–*pBac* or the *phsp–^i^hyPBase* transposase helper (200 or 300 ng/µL) was injected into WT embryos with the same set-up as described for *Ae. aegypti* injections, except for using pre-siliconized borosilicate needles (GB100F-10, Science Products GmbH, Germany) instead of quartz needles. In a third series of experiments, the two helper plasmids were combined at 200 ng/µL each with 500 ng/µL donor plasmid. G_0_ adults were individually (unless otherwise stated) crossed to WT flies, and G_1_ flies were screened for fluorescence. Segregation tests were conducted by outcrossing the transformants to WT flies, and transgenic lines were established from single G_1_-positive adults.

### 4.7. Copy Number Variation (CNV) and Linkage Analysis of Transgene Integrations by Droplet Digital PCR (ddPCR)

The number of genomic integrations of the transgene cassettes in *Ae. aegypti* was analyzed by ddPCR probing for the eGFP or DsRed marker genes. The reference gene was mEF1, a one-copy gene in the *Ae. aegypti* genome. ddPCR was performed with the BIO-RAD QX200 and Auto-DG System. The 20 µL CNV reactions contained 20–100 ng *Ae. aegypti* genomic DNA, 1x ddPCR Supermix for probes (BIO-RAD #1863010), 1x primer-probe mix target gene (FAM-labeled), 1x primer-probe mix reference gene (HEX-labeled), and 1U EcoRI (New England Biolabs; EcoRI cuts within the transgene constructs but not within the PCR amplicons). Droplets were generated with the Automated Droplet Generator. PCR cycling conditions (deep well block) were: 95 °C for 10 min, 40x [94 °C for 30 s, 60 °C for 1 min], 98 °C for 10 min, 4 °C hold. The ramp rate was 2 °C/s. Primer and probes were prepared and stored as a 20x primer-probe mix consisting of 18 µM each of forward and reverse primer and 5 µM probe (the final concentration of primers and probe in the reaction was 900 nM and 250 nM, respectively). Primers and probe used for target gene EGFP were: EGFP-for (P106) = 5′caaagaccccaacgagaagc, EGFP-rev (P108) = 5′gtccatgccgagagtgatcc, EGFP-probe = 5′ FAM-cgatcacatggtcctgctgg-BHQ1. Primers and probe used for target gene DsRed were: DsRed-for (P49) = 5′gatccacaaggccctgaagc, DsRed-rev (P50) = 5′gctccacgatggtgtagtcc, DsRed-probe = 5′ FAM-tcgttgtgggaggtgatgtc-BHQ1. Primers and probe used for reference gene mEF1 were: mEF-for (P63) = 5′tccggtttgcctacgatacc, mEF-rev (P64) = 5′actgggcagttgtactcacg, mEF-probe = 5′ HEX-tcgggaatgggtgaattgca-BHQ1.

The distribution of positive and negative droplets in each well was analyzed individually, and the threshold was corrected manually if necessary.

For linkage analysis, the CNV experiment was once conducted with restriction digest and once without, and the results were compared. If two transgene cassettes are linked on the same chromosome, the copy number obtained from the undigested reaction is approximately ½ of the digested reaction. Linkage analysis requires cautious preparation of genomic DNA to avoid shearing forces. Depending on the extent of shearing and the distance of the two integration sites, the value obtained from undigested DNA can converge towards the value of the digested sample.

Descriptions of plasmids used in this study are provided in Supplementary Table S9.

Plasmid cloning strategies of the plasmids used in this work that are not already published elsewhere are provided in Supplementary Methods in Supplementary File 1. Some of the precursor versions that the plasmids have been build from have already been published elsewhere [29,42,43,44,45,46,47,48,49].

Primer sequences are provided in Table S10. 

### 4.8. Analysis of Genomic Integration Sites by Inverse PCR

Genomic locations of the transgene constructs were determined by inverse plus nested PCR according to the following protocol: genomic DNA (600 ng) was digested with 4 U MspI in a 20 µL reaction for 1 hr at 37 °C. Digested DNA was immediately precipitated, pelleted, and re-dissolved in 50 µL TE buffer. The complete amount of MspI-digested DNA (50 µL) was used for the subsequent self-ligation reaction in 350 µL total volume containing 1x T4 Ligation Buffer and 2 µL T4 DNA ligase (NEB, 400,000 U/mL) overnight at 16 °C. Ligated DNA was precipitated and dissolved in 50 µL. A total of 3 µL DNA was PCR amplified in 20 µL containing 1x Phusion Flash High-Fidelity Polymerase Mastermix (Thermo Scientific F548S) and 500 nM of each primer. Cycling conditions for touchdown inverse PCR (iPCR) were: 98 °C for 10 s, 5x [98 °C for 1 s; Tm + 5 °C for 5 s, reduced by 2 °C per cycle; 72 °C for 1 min], 30x [98 °C for 1 s; Tm + 5 °C for 5 s; 72 °C for 1 min], 72 °C for 1 min, 12 °C hold. The annealing temperature was adjusted for each primer pair.

The iPCR reaction was either directly purified by agarose gel electrophoresis and sent to sequencing or diluted 1:100, and 1 µL used for the (semi-) nested PCR. (Semi-) nested PCR reactions were identical to the iPCR reactions. Cycling conditions for (semi-) nested PCR (nPCR) were: 98 °C for 10 s, 30x [98 °C for 1 s, Tm minus 5 °C for 5 s, 72 °C for 1 min], 72 °C for 1 min, 12 °C hold. Primers for probing the 3′ *piggyBac* integration site were mfs12 (5′CCTCGATATACAGACCGATAAAACAC)/P139 (5′CTTTTATCGAATTCCTGCAGC) (iPCR) and mfs34 (5′CGTACGTCACAATATGATTATCTTTCTAGG)/P139 (nPCR), and for probing the 5′ *piggyBac* integration site mfs10 (5′ACGACCGCGTGAGTCAAAATGACG)/mfs11 (5′ATCAGTGACACTTACCGCATTGACA) (iPCR) and mfs10/mfs31 (5′CGACTGAGATGTCCTAAATGCACAG) (nPCR). 

### 4.9. Transposition and Recombination Efficiency Calculation

Transposition or recombination efficiencies typically are defined as:

efficiency (%) = (number of independent transgenic events) × 100/(number of fertile G_0_ adults)

In our study, we calculated minimal transposition/recombination efficiencies as the number of obtained independent transgenic lines divided by the total number of adult G_0_ survivors of each injection. The actual transposition efficiency in several cases was higher than the numbers reported here for several reasons. All emerged G_0_ adults were counted for efficiency calculation, including sterile individuals; this was necessary to avoid bias in efficiency calculation between the individual backcross and the group backcross experiments, where sterile individuals would not have been detected. Moreover, not all potentially present independent integration events were detected because (i) only the individuals with CN = 1 were analyzed for their genomic integration site by inverse PCR. Thus, potentially different integration events in individuals with CN > 1 were not detected; (ii) only a subset of positive G_1_ offspring of most G_0_ families was individually backcrossed and analyzed, while the rest was group backcrossed and kept as backup; (iii) in some experiments with lots of positive G_0_ families not all families were further characterized (V19, V369, V370); and (iv) some G_1_ found dead in cages did not produce sufficient quality DNA for digital and inverse PCR. Finally, it has to be noted that individual backcrossing in V369 and V370 injections was only started at the G_3_ generation after two generations of group backcrosses of positive individuals. Not all positive G_1_ were used for backcrossing. Some integration events might have been lost.

### 4.10. Statistics

Differences in fitness parameters and transgenesis efficiencies between *phsp*–*pBac* and *phsp–^i^hyPBase* injections or helper plasmid and helper mRNA injections were analyzed with single factor ANOVA at alpha = 0.05.

## 5. Conclusions

*pBac* mRNA strongly increased the *pBac* transposition efficiencies in *Ae. aegypti* and *Ae. albopictus*. The use of mRNA probably circumvents inefficient transcription from *pBac* helper plasmids. In contrast, RMCE efficiencies could not be improved in the two tested *Aedes* species. Here, the limiting factor might not be the recombinase availability in the first place but rather the more complex reaction dynamics.

Based on the strong *pBac* transformation efficiency improvement observed in our study, it would be interesting to try *pBac* mRNA injections in other insect species, especially if low transformation efficiency is suspected to be caused by low transposase expression from a helper plasmid. Moreover, mRNA injections could also be tested for other transposases such as Hermes, Minos, or Hobo. We believe that the use of transposase mRNA might have the potential to make a change in the field of insect transformation.

## Figures and Tables

**Table 1:** Transformation efficiencies in *Aedes aegypti* using the standard *piggyBac* helper or ^i^hyPBase helper plasmids.

Exp. No	Helper Template	[Helper/Donor] (ng/µL)	Donor Construct	Donor Plasmid (Insert) Size (bp)	No. Injected Embryos	Larval Hatch Rate (%)	Adult Eclosion Rate (%)	Total No. G_0_ Families	% Fertile G_0_ Families	No. Transg. G_0_ Families	Total No. G_1_ Screened	No. Transg. Events	Transp. Eff. (%)
1	*pBac*	300/150	AH452 *	(5267) 8649	623	29.21	78.57	8	100.00	4	n.d.	4	2.80
2	*pBac*	200/500	V3	(3545) 6911	234	31.20	86.30	25	52.00	1	8830	1	1.25
3	*pBac*	400/600	V285	(5180) 8682	705	25.82	64.29	18	88.89	2	14,966	2	1.71
**4**	***pBac***	**300/500**	**V286**	**(5600) 9102**	**680**	**13.38**	**41.76**	**15**	**60.00**	**0**	**8772**	**0**	**0.00**
**5**	***pBac***	**160/185**	**V258**	**(6190) 9690**	**922**	**10.41**	**37.50**	**18**	**83.33**	**0**	**6631**	**0**	**0.00**
**6**	***pBac***	**400/600; 228/342**	**V258**	**(6190) 9690**	**599**	**24.21**	**34.48**	**11**	**100.00**	**0**	**18,721**	**0**	**0.00**
7	*pBac*	300/150	V257	(7088) 10,587	1113	42.59	76.37	26	100.00	2	93998	2	0.55
8	*pBac*	200/500	V19	(3630) 7163	257	18.29	63.83	9	100.00	2	5185	2	6.67
9	*pBac*	300/500	V19	(3630) 7163	192	33.85	61.54	11	63.64	0	2679	0	0.00
10	*pBac*	200/500	V19	(3630) 7163	399	13.53	61.11	13	46.15	0	1313	0	0.00
11	*pBac*	200/500	V19	(3630) 7163	462	14.72	48.53	7	100.00	2	1389	2	6.06
12	*pBac*	300/300	V368	(5835) 11,097	257	5.84	60.00	7	42.86	0	502	0	0.00
13	*pBac*	100/300	V368	(5835) 11,097	148	5.41	62.50	4	75.00	0	382	0	0.00
												avg	1.46
**14**	**^i^hyPB**	**100/200**	**V286**	**(5600) 9102**	**594**	**8.08**	**75.00**	**12**	**75.00**	**0**	**9382**	**0**	**0.00**
**15**	**^i^hyPB**	**200/200**	**V286**	**(5600) 9102**	**730**	**8.63**	**79.37**	**17**	**70.59**	**0**	**7604**	**0**	**0.00**
**16**	**^i^hyPB**	**200/200**	**V258**	**(6190) 9690**	**895**	**6.82**	**65.57**	**13**	**69.23**	**0**	**5199**	**0**	**0.00**
												avg	0.00

Shown are the injection data using the standard *phsp–pBac* helper plasmid (*pBac*) collected over six years and injections performed using the *phsp–^i^hyPBase* plasmid (^i^hyPB) containing the insect codon-based hyperactive *pBac* version. Injections 4, 14, and 15, as well as 5, 6, and 16 (printed in bold), were performed in parallel with the same donor plasmid and eggs from the same female cohort. “Injected embryos” represents the number of black embryos 24 h post-injection; “hatch rate” = no. larvae/no. black eggs (%); “eclosion rate” = no. adults/no. larvae (%); “no. transg. events” is the number of independent transposition events observed; “transp. eff.” = the minimal transposition efficiency, calculated as the number of independent transposition events/total number of G_0_ adults. The actual transposition efficiency can be higher because, in group backcrosses of G_0_ individuals, the number of infertile G_0_ is not known. Therefore, all G_0_ are included in the calculation; n.d. = not determined; avg = average; * this data has been published before in Häcker et al. 2017 [29].

**Table 2:** Transformation efficiencies in *Drosophila suzukii* with the standard *pBac* helper or ^i^hyPBase helper plasmids.

Exp. No.	Helper Template	[Helper/ Donor] (ng/µL)	Donor Construct	Donor Plasmid (Insert) Size (bp)	No. Injected Eggs	No. Hatched Larvae	Hatch Rate (%)	No. Fertile Adults	Fertile Eclosion Rate (%)	No. Transg. Lines	Transp. Eff. (%)
Ds 1	*pBac*	200/500	AH443	(9191) 12,576	75	n.d.	n.d.	25	n.d.	4	16.00
Ds 2	*pBac*	200/500	V220	(7865) 11,249	1601	231	14.43	65	28.14	1	1.54
**Ds 3**	***pBac***	**200/500**	**V221**	**(7867) 11,252**	**443**	**102**	**23.02**	**9**	**8.82**	**0**	**0.00**
Ds 4	*pBac*	200/500	V146	(7118) 10,503	481	173	35.97	20	11.56	2	10.00
Ds 5	*pBac*	200/500	V183	(7583) 10,968	753	305	40.50	43	14.10	5	11.63
Ds 6	*pBac*	200/500	V184	(9438) 12,823	640	167	26.09	17	10.18	0	0.00
Ds 7	*pBac*	200/500	V185	(8493) 11,878	802	346	43.14	53	15.32	6	11.32
Ds 8	*pBac*	200/500	V188	(10,347) 13,732	631	285	45.17	55	19.30	1	1.82
Ds 9	*pBac*	200/500	V213	(10,059) 13,443	538	107	19.89	27	25.23	0	0.00
Ds 10	*pBac*	200/500	V215	(8204) 11,589	626	173	27.64	29	16.76	1	3.45
Ds 11	*pBac*	200/500	V226	(8669) 12,054	410	51	12.44	9	17.65	1	11.11
Ds 12	*pBac*	200/500	V227	(10,524) 13,909	493	122	24.75	12	9.84	1	8.33
Ds 13	*pBac*	200/500	V228	(9113) 12,498	310	97	31.29	13	13.40	0	0.00
Ds 14	*pBac*	200/500	V250	(9578) 12,963	378	129	34.13	28	21.71	0	0.00
Ds 15	*pBac*	200/500	V229	(10,967) 14,352	339	92	27.14	26	28.26	1	3.85
Ds 16	*pBac*	200/500	V251	(11,433) 14,818	413	70	16.95	10	14.29	0	0.00
**Ds 17**	***pBac***	**200/500**	**V265**	**(9952) 13,337**	**520**	**124**	**23.85**	**32**	**25.81**	**0**	**0.00**
										avg	4.65
Ds 18	*pBac*	300/700	V229	(10,967) 14,352	1795	315	17.55	64	20.31	6	9.4
Ds 19	*pBac*	300/700	V251	(11,433) 14,818	1495	248	16.59	21	8.47	0	0.0
**Ds 20**	***pBac***	**300/700**	**V265**	**(9952) 13,337**	**582**	**238**	**40.89**	**77**	**32.35**	**5**	**6.49**
										avg	3.47
**Ds 21**	***pBac* + ^i^hyPB**	**200 + 200/500**	**V221**	**(7867) 11,252**	**1457**	**271**	**18.60**	**42**	**15.50**	**1**	**2.38**
Ds 22	*pBac* + ^i^hyPB	200 + 200/500	V222	(7861) 11,246	1898	401	21.13	34	8.48	0	0.00
Ds 23	*pBac* + ^i^hyPB	200 + 200/500	V223	(7865) 11,249	488	82	16.80	30	36.59	2	6.67
										avg	3.02
Ds 24	^i^hyPB	200/500	V209	(5538) 8922	1081	252	23.31	37	14.68	2	5.41
**Ds 25**	**^i^hyPB**	**200/500**	**V265**	**(9952) 13,337**	**98**	**32**	**32.65**	**8**	**25.00**	**0**	**0.00**
										avg	2.70

Shown are the injection data using the standard *phsp–pBac* helper plasmid (*pBac*) (exp. Ds 1–20) and injections performed using *phsp–^i^hyPBase* helper plasmid (^i^hyPB) (Ds 24, 25) or a combination of both (Ds 21–23). The injections for AH443 [18], V220–223 [30], V146, V183–V185, V188, V213, V215, V226–229, V250–251 [31], V209 [32], and V265 [33] were performed across more than 10 years. All injected eggs were counted. “hatch rate” = no. larvae/no. injected eggs (%); “fertile eclosion rate” = no. fertile G_0_ adults/no. hatched larvae (%); “no. transg. Lines” is the number of independent transgenic lines obtained (all G_0_ were backcrossed individually, except for injection Ds 18; here independent lines were identified from each family and confirmed by segregation analysis); “transp. eff.” = the transposition efficiency, calculated as the number of independent transgenic events/total number of fertile G_0_ adults; bold print highlights *phsp–pBac* and *phsp*–*^i^hyPBase* injections with the same donor plasmid; n.d. = not determined; avg = average.

**Table 3:** Transformation data in *Ae. aegypti* using the *phsp–pBac* helper plasmid or capped *pBac* mRNA as *transposase* source.

Exp. No	Helper Template	[Helper/Donor] (ng/µL)	Donor Construct	Donor Plasmid (Insert) Size (bp)	No. Injected Embryos	Hatch Rate (%)	Eclosion Rate (%)	Total No. G_0_ Families	No. Fertile G_0_ Families	No. Transg. G_0_ Families	Total No. G_1_ Screened	No. Transg. Events *	No. Transg. Events/G_0_	Transp. Eff. (%)
1	plasmid	300/150	AH452 ***	(5267) 8649	623	29.21	78.57	8	8	4	n.d.	4	1	2.80
2	plasmid	200/500	V3	(3545) 6911	234	31.20	86.30	25	13	1	8830	1	1	1.59
3	plasmid	400/600	V285	(5180) 8682	705	25.82	64.29	18	16	2	14,966	2	1	1.71
4	plasmid	300/500	V286	(5600) 9102	680	13.38	41.76	15	9	0	8772	0	n.a.	0.00
5	plasmid	160/185	V258	(6190) 9690	922	10.41	67.71	18	15	0	6631	0	n.a.	0.00
6	plasmid	400/600; 228/342	V258	(6190) 9690	599	24.21	58.62	11	11	0	18,721	0	n.a.	0.00
7	plasmid	300/150	V257	(7088) 10,587	1113	42.59	76.37	26	26	2	93,998	2	n.d.	0.55
8	plasmid	200/500	V19	(3630) 7163	257	18.29	63.83	9	9	2	5185	2	1	6.67
9	plasmid	300/500	V19	(3630) 7163	192	33.85	61.54	11	7	0	2679	0	n.a.	0.00
10	plasmid	200/500	V19	(3630) 7163	399	13.53	61.11	13	6	0	1313	0	n.a.	0.00
11	plasmid	200/500	V19	(3630) 7163	462	14.72	48.53	7	7	2	1389	2	1	6.06
**12**	**plasmid**	**300/300**	**V368**	**(5835) 11,097**	**257**	**5.84**	**60.00**	**7**	**3**	**0**	**502**	**0**	**n.a.**	**0.00**
**13**	**plasmid**	**100/300**	**V368**	**(5835) 11,097**	**148**	**5.41**	**62.50**	**4**	**3**	**0**	**382**	**0**	**n.a.**	**0.00**
													avg	1.49
14	mRNA	182/300	V96	(5278) 8813	113	11.50	46.15	6	3	1	370	4	4	66.67
15	mRNA	182/300	V97	(4307) 7841	298	5.03	73.33	11	4	2	514	7	≥2–≥5	63.64
16	mRNA	300/300	V370	(5870) 11,131	576	31.25	86.67	12	12	11	4388	>19	≥1	12.18
17	mRNA	300/300	V369	(6420) 11,684	520	16.35	80.00	6	6	5	2173	>8	≥1	11.76
18	mRNA	300/300	V19	(3630) 7163	527	8.92	48.94	28	17	10	1813	≥24	1–≥5	104.35 **
19	mRNA	100/300	V19	(3630) 7163	310	5.48	70.59	9	6	2	1195	≥7	≥3–≥4	58.33
20	mRNA	100/300	V19	(3630) 7163	646	1.61	80.00	7	7	3	1030	4	1–2	50.00
**21**	**mRNA**	**300/300**	**V368**	**(5835) 11,097**	**449**	**7.35**	**66.67**	**18**	**10**	**5**	**1014**	**≥ 12**	**≥2–4**	**54.55**
**22**	**mRNA**	**100/300**	**V368**	**(5835) 11,097**	**281**	**4.98**	**85.71**	**7**	**6**	**5**	**870**	**≥ 9**	**1–≥3**	**75.00**
													avg	49.02

Data from 13 injection experiments using the *phsp–pBac* helper plasmid over six years and nine injection experiments using *pBac* capped mRNA over five years are displayed. Exps. 1–13 are identical to the ones shown in Table 1. Exp. 12, 13, 21, and 22 (printed in bold) were performed in parallel with eggs from the same female cohort; injections 14 and 15 were performed in the wild type Orlando laboratory strain, and all other injections in the Higgs White Eye strain; “Injected embryos” represents the number of black embryos 24 h post-injection; “hatch rate” = no. larvae/no. black eggs (%); “eclosion rate” = no. adults/no. larvae (%); “no. transg. events” is the number of independent transposition events observed (multiple genomic integrations in one individual were counted as one event); “no. of transg. events/G_0_” = the maximum number of independent transposition events detected in a G_0_ founder individual as determined by ddPCR and iPCR analysis of positive G_1_. If the number is given as ‘≥ number,’ then only a subset of positive G_1_ was molecularly analyzed, and additional independent events might not have been detected; “transp. eff.” = the minimal transposition efficiency, calculated as the number of independent transgenic events/total number of G_0_ adults. The transposition efficiency in several families is assumed to be higher (see text); n.a. = not applicable; n.d. = not determined; avg = average; * for detailed numbers of all transgenic events identified in each G_0_ family and calculations see Tables S2 and S3; ** transposition efficiency of > 100% results from single G_0_ founder individuals producing more than one independent transposition event (as determined by ddPCR and iPCR); *** this data has been published before in Häcker et al. 2017 [29].

**Table 4:** Transformation data in *Ae. albopictus* using capped mRNA as the *pBac* source.

Exp. No	Helper Template	[Helper/Donor] (ng/µL)	Donor Construct	Donor Plasmid (Insert) Size (bp)	No. Injected Embryos	Hatch Rate (%)	Eclosion Rate (%)	Total No. G_0_ Families	No. Fertile G_0_ Families	No. Transg. G_0_ Families	Total G_1_ Screened	No. Transg. Events	No. Transg. Events/G_0_	Transf. Eff. (%)
1	mRNA	300/150	AH452 modified *	(5205) 7845	721	39.53	83.51	19	19	15	7049	≥15	≥1	6.3%

Displayed are data from one injection experiment using *pBac*-capped mRNA in *Ae. albopictus*. “no. injected embryos” = total number of injected embryos; “hatch rate” = no. larvae/total no of injected eggs (%); “eclosion rate” = no. adults/no. larvae (%); “no. transg. events” is the number of independent transposition events observed; “transp. eff.” = the minimal transposition efficiency, calculated as the number of independent transgenic events/total number of G_0_ adults. The actual transposition efficiency might be higher (see text). For more details on *Ae. albopictus pBac* transformation data, please see Table S5. * In this plasmid, the *D. melanogaster* PUbCFP cassette was replaced with an *Ae. aegypti* PUbeCFP cassette from pSL1180-HR-PUbeCFP (Addgene plasmid 47917).

**Table 5:** phiC31-RMCE injection data in *Ae. aegypti* using *phsp–phiC31* helper plasmid or capped phiC31 mRNA.

Exp. No	Helper Template	[Helper/Donor] (ng/µL)	Donor Construct	Donor Plasmid (Insert) Size (bp)	No. Injected embryos	Hatch Rate (%)	Eclosion Rate (%)	Total No. G_0_ Families	No. Fertile G_0_ families	No. Transg. G_0_ Families	Total G_1_ Screened	No. Transg. Events	Recomb. Eff. (%)
1	*phsp-phiC31*	150/300	V101	(4404) 8216	510	3.14	75.00	9	6	0	1690	0	0
2	*phsp-phiC31*	150/300	V101	(4404) 8216	237	7.59	61.11	5	5	0	2851	0	0
3	mRNA	150/300	V101	(4404) 8216	502	4.38	59.09	11	4	0	534	0	0
4	mRNA	150/300	V101	(4404) 8216	335	3.28	54.55	4	2	0	458	0	0
5	mRNA	150/300	V101	(4404) 8216	361	3.32	83.33	7	4	1 *	318	1	10
6	*phsp-phiC31*	300/500	V101	(4404) 8216	443	2.71	91.67	9	4	0	1059	0	0
7	*phsp-phiC31*	300/500	V101	(4404) 8216	302	13.25	77.50	11	9	2	6068	2	6.45
8	mRNA	300/500	V101	(4404) 8216	412	2.18	100.00	9	9	0	6250	0	0
9	mRNA	300/500	V101	(4404) 8216	248	13.71	73.53	10	6	0 **	3650	0	0

Data from comparative injection experiments are displayed at two different helper/donor concentrations into the same landing site line; “no. injected embryos” represents the number of black embryos 24 h post-injection; “hatch rate” = no. larvae/no. black eggs (%); “eclosion rate” = no. adults/no. larvae (%), “no. transg. events” is the number of independent recombination events observed; “recomb. eff.” is the minimal recombination efficiency, calculated as the number of transgenic events/total number of G_0_ adults. The recombination efficiency can be higher, as in group backcrosses of G_0_, the number of infertile G_0_ is unknown. All positive individuals obtained showed the RMCE phenotype. * One additional family with transient donor phenotype; parental phenotype in next generation; ** one family with transient donor phenotype; parental phenotype in next generation.

**Table 6:** Cre-RMCE injection data in *Ae. aegypti* using *phsp–Cre* helper plasmid or capped Cre mRNA.

Exp. No	Helper Template	[Helper/Donor] (ng/µL)	Donor Construct	Donor Plasmid (Insert) Size (bp)	No. Injected embryos	Hatch Rate (%)	Eclosion Rate (%)	Total No. G_0_ Families	No. Fertile G_0_ Families	No. Transg. G_0_ Families	Total G_1_ Screened	No. Transg. Events	Recomb. Eff. (%)
1	*phsp–Cre*	450/350	V20	(1297) 4997	765	18.69	57.34	13	12	2 *	9283	2	2.44
2	*phsp–Cre*	450/350	V20	(1297) 4997	165	6.67	72.73	4	1	0	390	0	0
3	*phsp–Cre*	450/350	V20	(1297) 4997	397	1.26	80.00	3	1	0	137	0	0
4	mRNA	450/350	V20	(1297) 4997	413	10.90	73.33	4	1	1 **	2419	1	3.03
5	mRNA	450/350	V20	(1297) 4997	230	5.22	58.33	3	3	0	996	0	0
6	mRNA	450/350	V20	(1297) 4997	348	5.75	80.00	12	9	0	4926	0	0

Data from comparative injection experiments are displayed at two different helper/donor concentrations into the same landing site line; “no. injected embryos” represents the number of black embryos 24 h post-injection; “hatch rate” = no. larvae/no. black eggs (%); “eclosion rate” = no. adults/no. larvae (%); “no. transg. Events” is the number of independent recombination events observed; “recomb. Eff.” Is the minimal recombination efficiency, calculated as the number of transgenic events/total number of G_0_ adults. The recombination efficiency can be higher, as in group backcrosses of G_0_, the number of infertile G_0_ is unknown. * One integration, one RMCE phenotype; ** integration phenotype.

## Data Availability

All data generated or analyzed during this study are included in this published article and its Supplementary Information Files.

## Supplementary Materials

The supporting information can be downloaded at https://www.mdpi.com/article/10.3390/ijms242015155/s1.
