# Supplementary material for: Improved piggyBac Transformation with Capped Transposase mRNA in Pest Insects
Source: Int J Mol Sci. 2023 Oct 13;24(20):15155. doi: 10.3390/ijms242015155 (PMC10606561; doi:10.3390/ijms242015155)
Supplement: Supplementary file 1 [file ijms-24-15155-s001.zip › Suppl file 1_edit.pdf]

Figure S1

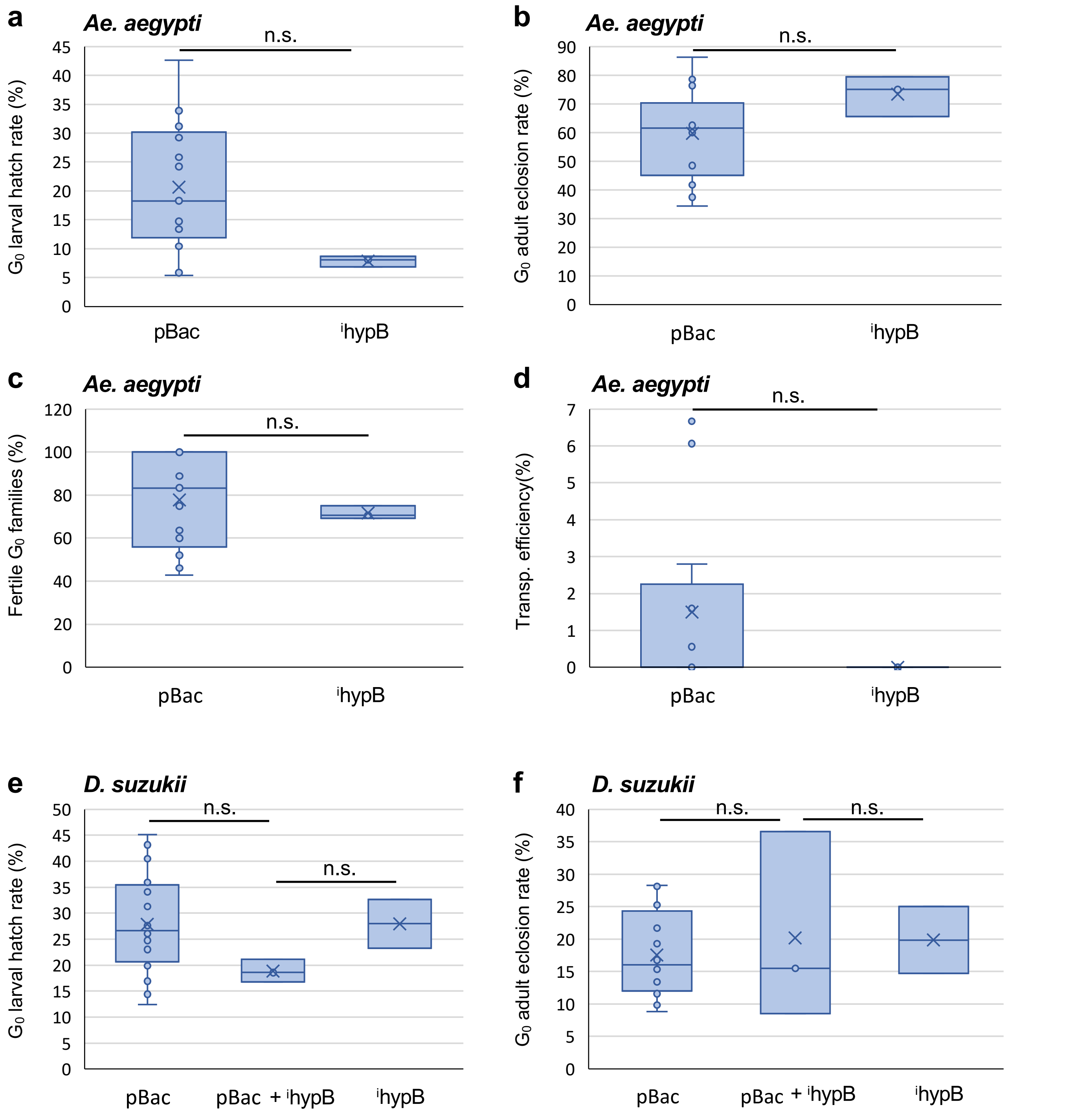

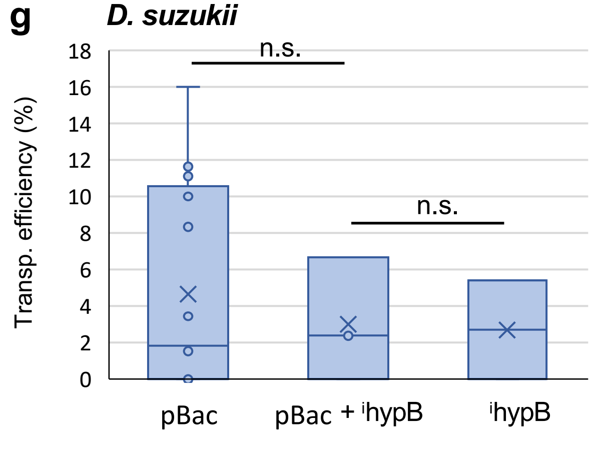

Effect of *phsp-pBac* helper plasmid and *phsp-^{i}hyPBase* helper plasmid in *Ae. aegypti* and *D. suzukii*. Box and whisker plots (exclusive median) are displayed on the data presented in Tables 1 and 2 for *Ae. aegypti* larval hatch rate (**a**), G_0 adult eclosion rate (**b**), G_0 fertility (percentage of fertile G_0 families in each experiment) (**c**), and the minimal transposition efficiency (%) (**d**), as well as *D. suzukii* larval hatch rate (**e**), G_0 adult eclosion rate (**f**),) and the minimal transposition efficiency (%) (**g**). The horizontal line represents the median, the cross (x) represents the mean; n.s. = no significant difference ($p > 0.05$, single factor ANOVA).

Figure S2

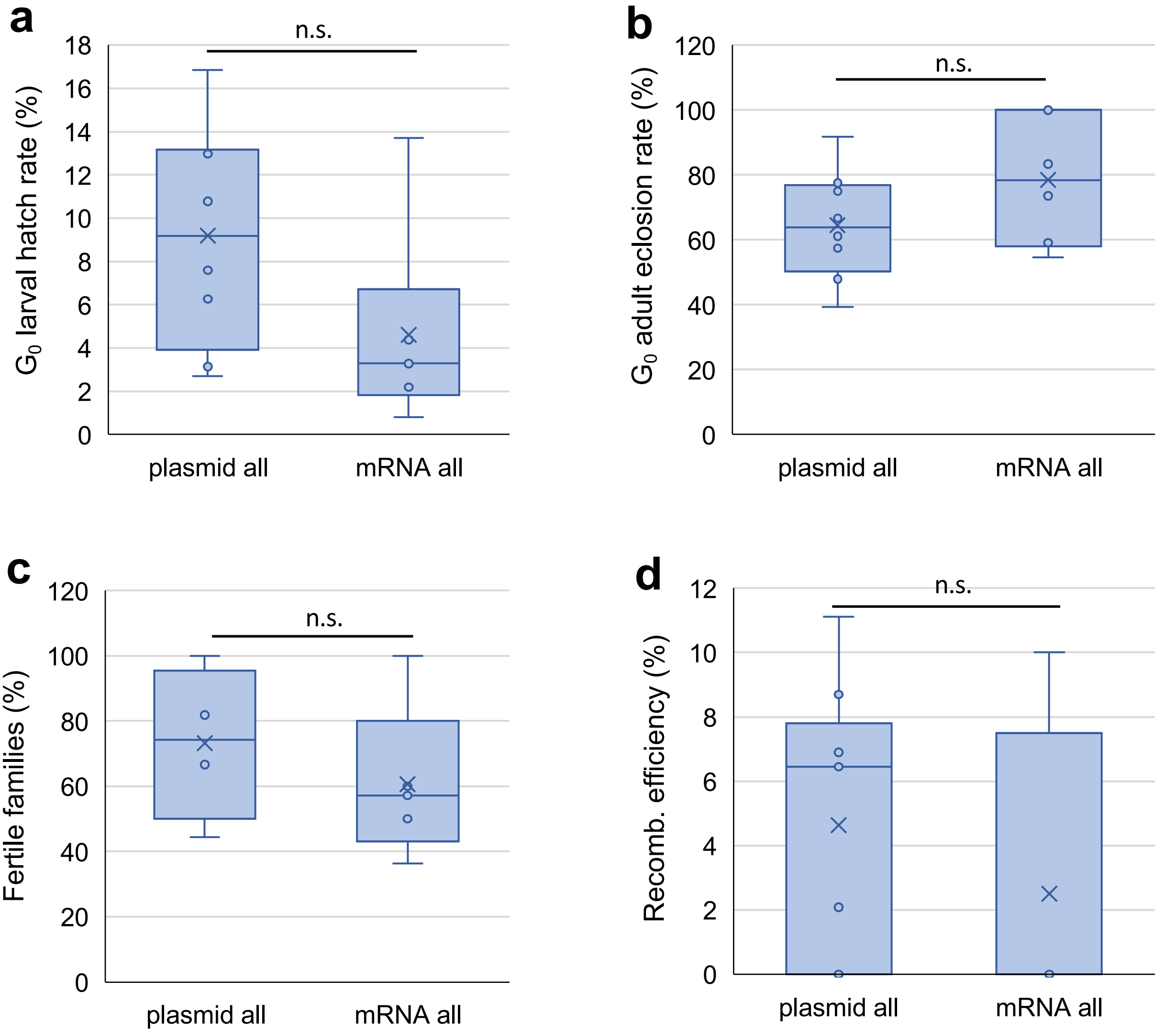

Effect of *phsp-phiC31* helper plasmid and phiC31 mRNA injections in *Ae. aegypti* on larval hatch rate (**a**), G_0 adult eclosion rate (**b**), G_0 fertility (**c**), and the minimal recombination efficiency (%) (**d**). Results from injections at low (150/300 ng/µl, exp. 1-5, Table 5) and high (300/500 ng/µl, exp. 6-9, Table 5) helper/donor concentrations were combined for data analysis ("plasmid all" and "mRNA all". Box and whisker plots (exclusive median) are displayed in the data presented in Table 4. The horizontal line represents the median, the cross represents the mean; n.s. = no significant difference ($p > 0.05$, single factor ANOVA).

Supplementary Methods

Plasmid cloning strategies

For construction of plasmid **V19** (*pB_attP_3xP3-EGFP_attPrev*), a 1230 bp fragment containing *3xP3-EGFP* was PCR-amplified from plasmid **AH459** (*pSL_FRT_3xP3-EGFP-SV40_FRT3)* [29] using primers P72, P73; a 310 bp fragment containing the *attP* site was PCR-amplified from plasmid #1286 (*pTA-attP*) [42] using primers P74, P75. Plasmid V1 (*pB_fa_attP(234))* was cut with AflII and NcoI, and the 5620 bp fragment retrieved. The two PCR fragments were ligated with the 5620 bp V1 fragment via Gibson assembly (Gibson Assembly Master Mix, New England Biolabs Inc, USA).

For construction of plasmid **V1**, the BglII and AflII cut vector backbone of the *piggyBac* vector **#1200** (*pB_fa_PUb-DsRed*) [43] ("long" pBac ends)) was ligated to the BglII/AflII cut *attP220* fragment generated by PCR on plasmid #1286 *pTA-attP* [42] using the primers MFS_1 and MFS_2.

V96 (*pBac_AaePub_DsRed.T3_SV40*) construction: Plasmid V25 (*pBac_fab_attP/220_PUb_DsRed-SV40*) was digested with Bsp119I and Bsu36I and the 5889 bp fragment retrieved. A 1418 bp *AaePUb* promoter fragment (AAEL003877 [44]) was PCR-amplified from HWE genomic DNA with primers P202 and P203. The DsRed-SV40 fragment (1551 bp) was PCR-amplified from V25 with primers P198 and P199. The PCR products were ligated with the V25 5889 bp backbone via Gibson assembly.

P97 (*pBac_AeUbl40_DsRed.T3_SV40*) construction: Plasmid V25 (*pBac_fab_attP/220_PUb_DsRed-SV40*) was digested with Bsp119I and Bsu36I and the 5889 bp fragment retrieved. The *AaeUbl40* promoter (AAEL006511 [44]) was PCR-amplified from HWE genomic DNA with primers P204 and P205. The DsRed-SV40 fragment (1551 bp) was PCR-amplified from V25 with primers P198 and P199. The PCR products were ligated with the V25 5889 bp backbone via Gibson assembly.

Plasmid **V25**: A 2995 bp *attP220_PUb-DsRedT3* fragment was PCR-amplified from #1425 [45] with primers MFS1 and P95 and digested with BglII and NotI. Plasmid #1200 [43] was digested with BglII and NotI and the backbone fragment ligated with the digested PCR product. The new fragment replaces the *PUb_DsRed* from #1200.

For **V101** (*pSL_attB_3xP3DsRed-FRT3_3'pB_3xP3-FRT-AmCyan_attBrev*) construction a 470 bp attB fragment was PCR-amplified (P298, P299) from #1285 [42], AflII digested, and ligated into the AflII-digested and dephosphorylated plasmid V94. *AttB* was verified to be in the reverse orientation via sequencing.

V94 (*pSL_attB_3xP3DsRed_3'pB_3xP3-FRT-AmCyan*) cloning: M727 (*p_3xP3_FRT_AmCyan* [46] was linearized via NheI digest. A 475 bp *attB* fragment (PCR-amplified from plasmid #1285 [42] (P228, P229), a 1250 bp *3xP3-DsRed* fragment (PCR-amplified from plasmid AH452 [29] (P230, P231), and a 1150 bp *3'pBac* fragment (PCR-amplified from plasmid #1200 [43] (P232, P233), were ligated in this order into the linearized plasmid M727 by Gibson assembly.

For construction of plasmid **V257** (*pBXLII_3XP3-FRT-ECFP_TREhs43_AlhidAla2_SV40_attP220*) the plasmid AH377 [17] was cut with AscI. Plasmid V256 (*pSL_fa_TREhs43_AlhidAla2-SV40-attP220*) was cut with AscI. The *TREhs43_AlhidAla2_SV40_attP220* fragment was retrieved and ligated into the cut AH377 and checked for orientation.

V256 was constructed by cutting plasmid #1403 (*pSL_TREhs43- SV40_attP220*) [45] with MluI and SalI. The fragment *Alhid*^{Ala2} was amplified from plasmid **M158**/ #1428 (*pSL_fa_TREhs43-AlhidAla2-SV40-attP235_fa*) [45] using primers P706 and P707. The fragment was then cloned into the cut vector #1403 via Gibson assembly.

Plasmid cloning strategies (continued)

For construction of plasmid **V258** (*pXLBacII_3xP3_DsRED.T3_SV40_AeaSry-α_tTA_SV40*) the plasmid **V257** was cut with NcoI and BglII. Vector V236 (*pBacXLII_attP_AeaPub_DsRED.T3_SV40_AeaSry-α_tTA_SV40*) was cut with NcoI and BglII and the fragment *DsRed.T3_SV40_AeaSry-α_tTa-SV40* was ligated into the cut V257.

V236 was cloned by opening plasmid **V113** (*pBXLII_AePUbDsReD.T3*) with FseI. A *AeaSry-α_tTA_SV40* fragment was PCR-amplified from **V233** using P633 and P634 and ligated with the opened V113 via Gibson assembly.

For cloning of **V113**, the *Ae. aegypti Poly-Ubiquitin* promoter region (AAEL003877 [44]) was PCR-amplified from genomic DNA of the Higgs White Eye (HWE) strain using primers P252 and P253, and cloned into plasmid #1425 [45] cut with Bsp119I and BstI via Gibson assembly.

V233 (*pSLaf_AeaSry-α_tTA_SV40*): plasmid #1215 (*pSLaf_tTA-SV40_af*) [45] was cut with XbaI and ligated via Gibson assembly with the *AeaSry-α* promoter that was PCR-amplified from *Ae. aegypti* HWE genomic DNA using P629 and P630

V285 (*pBXLII_3XP3_DsRed_SV40_Aae-b2tubpro-540bp_tTA_SV40*) was cloned by cutting plasmid V258 with SacII and BglII. The fragment *SacII_Aae-b2tub540* was amplified from V217 (*pBXLBII-AePUbDsRED_Aeb2tub540_tTA*) using primers P799 and P800. The fragment *tTA_SV40_BglII* was amplified from V258 using primers P801 and P802. The fragments were then ligated to the 6537 bp fragment of V258 via Gibson assembly.

V286 (*pBXLII_3XP3_DsRed_SV40_Aae-b2tubpro-960bp_tTA_SV40*) was cloned by cutting plasmid V258 with SacII and BglII. The fragment *SacII_Aae-b2tub960* was amplified from V216 (*pBXLII-AePUbDsRED_Aeß2tub960_tTA*) using primers P799 and P800. The fragment *tTA_SV40_BglII* was amplified from V258 using primers P801 and P802. The fragments were then ligated to the 6537 bp fragment of V258 via Gibson assembly.

V216: The 960 bp *Ae. aegypti beta 2-tubulin* promoter region was PCR-amplified from genomic DNA (HWE) using primers P519 and P562, Bsu36I and NheI digested, and ligated into the #1215 plasmid (*pSLaf_tTA-SV40_af*) [45] opened with Bsu36I and NheI.

V217: The 540 bp *Ae. aegypti beta 2-tubulin* promoter region was PCR-amplified from genomic DNA (HWE) using primers P561 and P562, Bsu36I and NheI digested, and ligated into the #1215 opened with Bsu36I and NheI.

V301 (*pSL_attB_3xP3-ECFP-SV40_TREhs43_AeIMP2-SV40_attBrev*) was constructed by Gibson ligation of a 1.43 kb *3xP3-ECFP* PCR product from AH352 (pBXLII-3xP3-ECFP) (P870, P871) into the 5.3 kb fragment of V109 digested with Bpu10I and NheI.

V109 (*pSL_attB_3xP3DsRed-SV40_TREhs43_AeIMP2-SV40_attBrev*) was constructed by Gibson ligation of a 1.656 kb *attB-3xP3-DsRed* PCR product from V101 (P780, P781) into V107 linearized with HindIII.

V107 (*pSL_TREhs43_AeIMP2-SV40_attBrev*) was constructed by Gibson ligation of a 235 bp *attBrev* PCR product from #1285 [42] (primers P777, P778) into V237 digested with BsrGI and EcoRI (4493 bp).

V237 (*pSL_fa_TREhs43_AeaIMPv2_SV40*) construction: the plasmid #1403 [45] was double digested with MluI and SalI. The 4,342 bp product was extracted and ligated using Gibson assembly with *AeaIMPv2* (*Ae. aegypti IAP inhibitor Michelob x like protein* (*IMP*) version 2, Häcker et al. unpublished) that was PCR amplified from HWE cDNA using P635 and P636.

Plasmid cloning strategies (continued)

V303 (*pSL_attB_3xP3-ECFP-SV40_TREhs43_AeMx2-SV40_attBrev*) was constructed by Gibson ligation of a 1.43 kb *3xP3-ECFP* PCR product from AH352 (P870, P871) into the 5.3 kb fragment of V110 digested with Bpu10I and NheI.

V110 (*pSL_attB_3xP3DsRed-SV40_TREhs43_AeIMP2-SV40_attBrev*) was constructed by Gibson ligation of a 1.656 kb *attB-3xP3-DsRed* PCR product from V101 (P780, P781) into V108 linearized with HindIII.

V108 (*pSL_TREhs43_AeMbxv2-SV40_attBrev*) was constructed by Gibson ligation of a 235 bp *attBrev* PCR product from #1285 [42] (primers P777, P778) into V238 digested with BsrGI and EcoRI (4493 bp).

V238 (*pSL_fa_TREhs43_Mbxv2_SV40*): plasmid #1403 [45] was double digested with MluI and SalI. The 4,342 bp product was extracted and ligated using Gibson assembly with *AeaMxv2* (*Ae. aegypti Michelob X* version 2*,* Häcker et al. unpublished), that was PCR amplified from *Aedes aegypti* cDNA from a Cayman Island strain using P637 and P638.

For construction of **V368** (*pBXLII_attB_3xP3DsRed-AaeNos-tTA*) plasmid V341 (*pSL_attB_3xP3DsRed-AaeNos-tTA*) was digested with AflII and FseI; plasmid #1204 (*pB_faf_sl2-tTA-SV40_a_PUb-DsRed)* was digested with AflII, FseI and MfeI. The 4896 bp fragment of V341 was ligated with the 6209bp fragment of #1204.

#1204 was constructed by opening the plasmid #1200 [43] with AscI and ligating the 3390 bp *sl2-tTA-SV40* fragment obtained by AscI digest of #1210 (pSLaf_*sl2-tTA-SV40*_af) [47].

V341 was cloned by PCR-amplifying a 450 bp *attB* fragment from #1285 (*pTA-attB*) [42] using primers P1130 and P229; a 1250 bp *3xP3DsRed* fragment was PCR-amplified from AH452 [29] with primers P230 and P1133; A 3043 bp *AaeNos-tTA* fragment was PCR-amplified from V231 (*pSLaf_AeaNanos_tTA_SV40*) with primers P1137 and P1138; all 3 fragments in this order were ligated into plasmid #1299 [48] opened with KpnI and XhoI via Gibson assembly.

V231 was cloned by cutting plasmid #1215 [45] with XbaI and ligated with the *Ae. aegypti Nanos* promoter that was PCR-amplified from HWE genomic DNA using P_625 and P_626.

For construction of **V369** (*pBXLII_attB_3xP3DsRed-AaeExu-tTA*) plasmid V342 (*pSL_attB_3xP3DsRed-AaeExu-tTA*) was first digested with AflII and the 3103 bp and 5537 bp fragments retrieved, followed by a digest of the 5537 bp fragment with FseI. Digestion of #1204 with AflII, FseI and MfeI. Ligation of 3103 bp fragment of the first V342 digest (AflII-3103 bp), the 2384 bp fragment of the second V342 digest (AflII and FseI-2384bp) and AflII/FseI-fragment of #1204 (6209bp).

V342 was cloned by PCR-amplifying a 450 bp *attB* fragment from #1285 [42] using primers P1130 and P229; a 1250 bp *3xP3DsRed* fragment was PCR-amplified from AH452 with primers P230 and P1134; A 3636 bp *AaeExu-tTA* fragment was PCR-amplified from V232 (*pSLaf_AeaExu_tTA_SV40*) with primers P1139, P1138; all 3 fragments in this order were ligated into plasmid #1299 [48] opened with KpnI and XhoI via Gibson assembly.

V232 was cloned by cutting plasmid #1215 with XbaI and ligated with AAEL010097 (*Aae-exuperantia*) promoter that was PCR-amplified from plasmid Pp#593 [49] using P627 and P628.

For construction of **V370** (*pBXLII_attB_3xP3DsRed-AaeSrya-tTA*) plasmid V343 (*pSL_attB_3xP3DsRed-AaeSrya-tTA*) was digested with AflII and FseI; plasmid #1204 was digested with AflII, FseI and MfeI. Ligation of the 4930 bp fragment of V343 and the 6209bp fragment of #1204.

V343 was cloned by PCR-amplifying a 450 bp *attB* fragment from #1285 [42] using primers P1130 and P229; a 1250 bp *3xP3DsRed* fragment was PCR-amplified from AH452 with primers P230 and P1135; A 3083 bp *AaeSrya-tTA* fragment was PCR-amplified from V233 with primers P1171, P1138; all 3 fragments in this order were ligated into plasmid #1299 [48] opened with KpnI and XhoI via Gibson assembly.

Plasmid cloning strategies (continued)

To generate the **modified AH452** construct (*pXL-BACII_FRT_3xP3DsRed_FRT3_loxN-PUbeCFP-lox2272*), the *D. melanogaster PUbCFP* cassette from AH452 [1] was replaced with an *Ae. aegypti PUb-eCFP* cassette from *pSL1180-HR-PUbeCFP* (Addgene plasmid 47917). One fragment from the AH452 plasmid was amplified using primers Vector_F and Vector_R, and a second fragment was amplified using primers SG_frag_2_Forward and SG_frag_2_Reverse. A third fragment containing the *Ae. aegypti PUb-eCFP* cassette was amplified from *pSL1180-HR-PUbeCFP* using primers SG_frag_1_Forward and SG_frag_1_Reverse. Bold letters in SG_frag_1_Forward and Vector_R, in SG_frag_1_Reverse and SG_frag_1_Forward, in Vector_R and SG_frag_1_Reverse, and Vector_F and SG_frag_2_Reverse represent complementary sequences. The complementary ends of each fragment were then annealed in a single reaction using an In-Fusion Cloning Kit (Takara, Ann Arbor, MI, USA).

References:
17. Horn C., Handler A.M. Site-specific genomic targeting in *Drosophila*. *Proceedings of the National Academy of Sciences, USA* **2005**, *102*, p. 12483-8.
29. Häcker I., Harrell II R.A., Eichner G., Pilitt K.L., O'Brochta D.A., Handler A.M., et al. Cre/lox-recombinase-mediated cassette exchange for reversible site-specific genomic targeting of the disease vector, *Aedes aegypti*. *Sci Rep* **2017**, *7*, p. 43883.
42. Groth A.C., Olivares E.C., Thyagarajan B., Calos M.P. A phage integrase directs efficient site-specific integration in human cells. *Proceedings of the National Academy of Sciences, USA* **2000**, *97*, p. 5995-6000.
43. Scolari F., Schetelig M.F., Bertin S., Malacrida A.R., Gasperi G., Wimmer E.A. Fluorescent sperm marking to improve the fight against the pest insect *Ceratitis capitata* (Wiedemann; Diptera: Tephritidae). *N Biotechnol* **2008**, *25*, p. 76-84.
44. Anderson M.A., Gross T.L., Myles K.M., Adelman Z.N. Validation of novel promoter sequences derived from two endogenous ubiquitin genes in transgenic Aedes aegypti. *Insect Mol Biol* **2010**, *19*, p. 441-9.
45. Schetelig M.F., Handler A.M. Strategy for enhanced transgenic strain development for embryonic conditional lethality in *Anastrepha suspensa*. *Proc Natl Acad Sci USA* **2012**, *109*, p. 9348-53.
46. Schetelig M.F., Handler A.M. A transgenic embryonic sexing system for *Anastrepha suspensa* (Diptera: Tephritidae). *Insect Biochem Mol Biol* **2012**, *42*, p. 790-5.
47. Schetelig M.F., Caceres C., Zacharopoulou A., Franz G., Wimmer E.A. Conditional embryonic lethality to improve the sterile insect technique in *Ceratitis capitata* (Diptera: Tephritidae). *BMC Biol* **2009**, *7*, p. 4.
48. Horn C., Wimmer E.A. A versatile vector set for animal transgenesis. *Development Genes and Evolution* **2000**, *210*, p. 630-7.
49. Akbari O.S., Papathanos P.A., Sandler J.E., Kennedy K., Hay B.A. Identification of germline transcriptional regulatory elements in Aedes aegypti. *Sci Rep* **2014**, *4*, p. 3954.
